# First-line anti-BCMA CAR-T cell therapy in a fragile patient with biclonal gammopathy and giant plasma cell tumor multiple myeloma with multiple comorbidities: a case report

**DOI:** 10.3389/fimmu.2025.1564774

**Published:** 2025-04-16

**Authors:** Yi Fang, Weiwei Lin, Lijing Shen, Beiwen Ni, Minyue Zhang, Yongmei Cai, Yi Zhou, Jian Hou

**Affiliations:** ^1^ Department of Hematology, Renji Hospital, School of Medicine, Shanghai Jiao Tong University, Shanghai, China; ^2^ Department of Clinical Laboratory, Renji Hospital, School of Medicine, Shanghai Jiao Tong University, Shanghai, China

**Keywords:** multiple myeloma, biclonal gammopathy, chimeric antigen receptor T cells, first-line therapy, case report

## Abstract

**Background:**

Chimeric antigen receptor T (CAR-T) cells targeting B-cell maturation antigen (BCMA) have been used as an effective therapy against relapsed/refractory multiple myeloma (MM). However, the relapse rates in these patients are still high, which may be related to the poor quality of T cells after multiple chemotherapies. The case reported here demonstrated the effectiveness and safety of first-line anti-BCMA CAR-T cell therapy for high-risk MM patients, even in frailty with multiple comorbidities.

**Case presentation:**

A 75-year-old woman was diagnosed with biclonal gammopathy and high-risk MM with extramedullary mass in the right caput femoris. The patient was fragile with multiple comorbidities, including pneumonia, left lower limb deep venous thrombosis, and epilepsy secondary to cerebral hemorrhage. Considering the patient’s fragility and comorbidities, commercial equecabtagene autoleucel, a fully human anti-BCMA CAR-T cells, as first-line CAR-T cell therapy, was proposed and accepted by the patient and her family. After one cycle of bortezomib, cyclophosphamide, and dexamethasone (VCD) regimen), she reached very good partial response (VGPR). Then her leukapheresis was performed, and the harvested cells were sent to the manufacturer for preparation. After lymphodepletion was performed using fludarabine and cyclophosphamide (FC) chemotherapy, her equecabtagene autoleucel was transfused. On day 21 after infusion, she achieved stringent complete remission (sCR) with minimal residual disease (MRD) negativity without severe toxicity. The CAR-T cells/CD3^+^ T cell ratio gradually increased, reaching a maximum of 54.97% on day 14, and gradually decreased, remaining at 0.03% on the 153rd day. The patient received right hip replacement plus pelvic lesion curettage 7 months after CAR-T transfusion due to pain in her right hip, but no MM cells were found in postoperative pathology. Hitherto, her deep remission persisted for 12 months without any maintenance therapy.

**Conclusion:**

First-line anti-BCMA CAR-T cell therapy is effective and safe for high-risk MM patients, even in fragile patients with multiple comorbidities.

## Introduction

Multiple myeloma (MM) is an incurable hematological malignancy characterized by the proliferation of abnormal clonal plasma cells in the bone marrow, leading to destructive bone lesions, renal injury, anemia, and hypercalcemia (1). High-risk factors in newly diagnosed MM include Revised International Staging System (R-ISS) stage III, extramedullary disease, circulating plasma cells, cytogenetic abnormalities (del(1p32), t(4;14), t(14;16), t(14;20), del(17/17p)/TP53 mutation, 1q21 gain/1q21 amplification, and MYC translocation), and a high-risk gene expression profile. Biclonal gammopathy is rare, occurring in fewer than 5% of MM patients, and is associated with poor early treatment response and high risk of early mortality reported in these patients (2, 3). In real-world settings, one third of MM patients are fragile, a condition identified as an independent risk factor for poor overall survival (OS) (4, 5). Elderly, frail patients may neither be able to tolerate intensive, long-term chemotherapy nor be suitable candidates for autologous stem cell transplantation, which limits treatment options and can negatively impact prognosis (6).

The first-line standard therapy for MM includes proteasome inhibitors and oral immunomodulators combined with dexamethasone (1, 4). On the other hand, adding daratumumab to the regimen is an alternative to high-risk patients (7), with a median progression-free survival (PFS) of about 41 months (1). However, the survival outcomes of these high-risk MM patients are still poor (8). Chimeric antigen receptor T (CAR-T) cells have successfully treated several hematologic malignancies. B-cell maturation antigen (BCMA) is the most selectively expressed receptor on MM cells and is one of the most promising therapeutic targets for MM (9–11). Equecabtagene autoleucel, a fully human CAR-T cell therapy targeting BCMA, has achieved remarkable results in treating relapsed/refractory MM patients (12–14).

Here we present the case of a 75-year-old Chinese woman with rare biclonal MM, classified as high risk due to an extramedullary mass, and fragile with multiple comorbidities, who was successfully treated with equecabtagene autoleucel as first-line therapy. She achieved minimal residual disease (MRD)-negative stringent complete remission (sCR) without severe toxicity and maintained deep remission for 12 months at the time of manuscript submission, without any maintenance therapy. This case report provides preliminary clinical evidence supporting the advancement of CAR-T cell therapy as a first-line treatment for frail patients with high-risk MM.

## Case report

**Figure 1:**
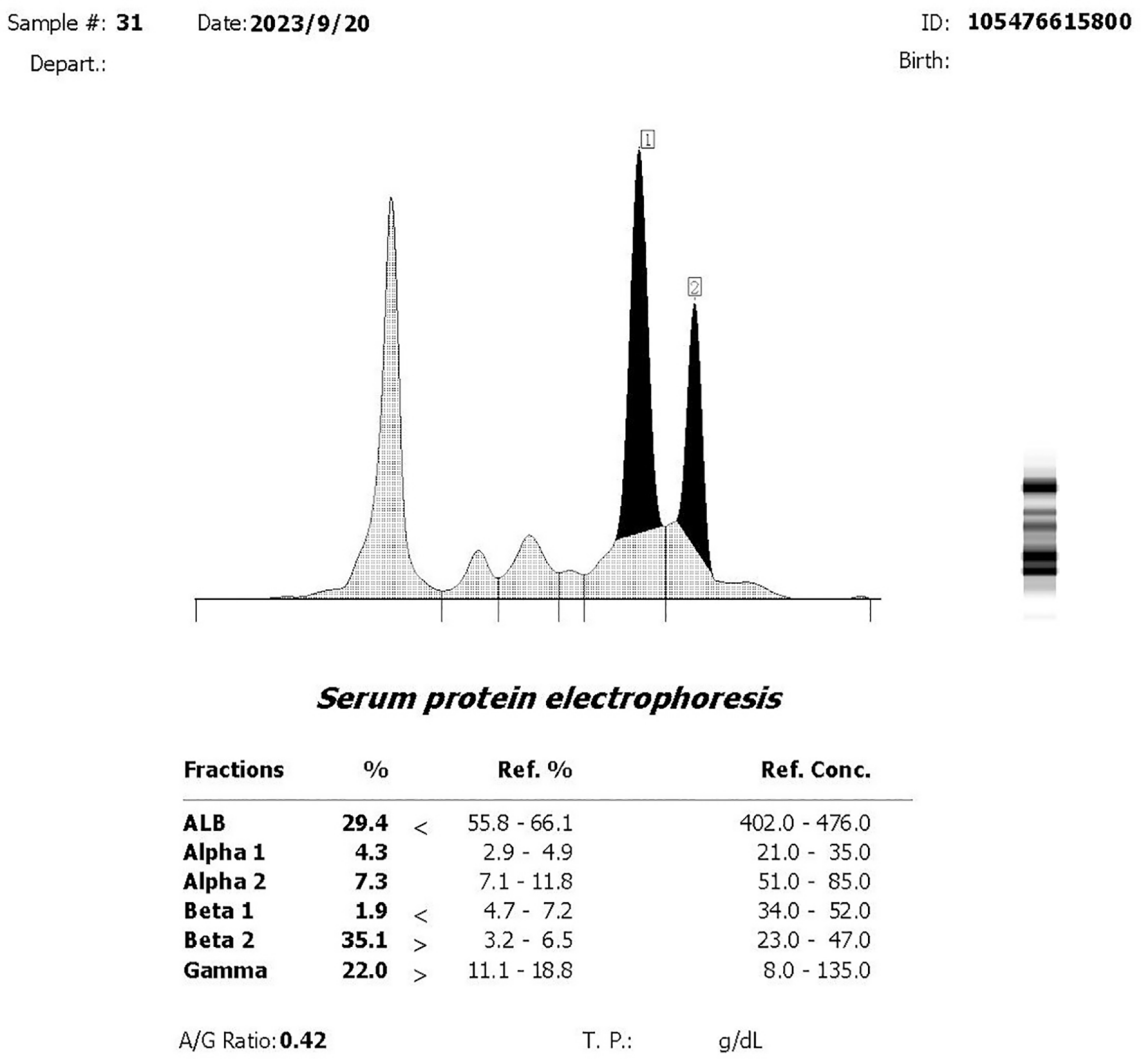
Immunofixation electrophoresis.

**Figure 2:**
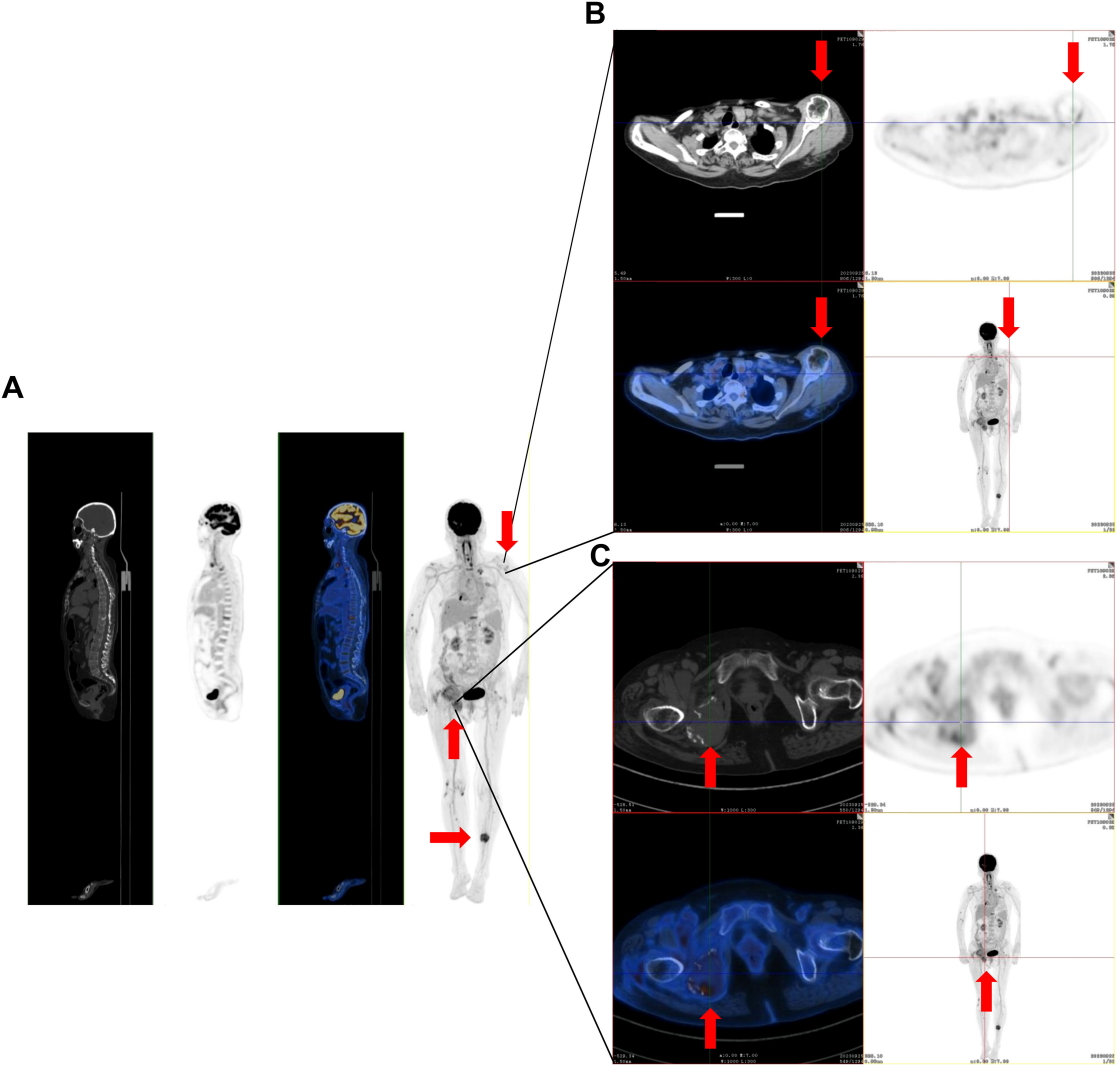
The representative positron emission tomography (PET)-CT images of multiple-site bone destruction and extramedullary mass. **(A)** PET-CT imaging demonstrates multiple regions of reduced bone density in the skull, multiple vertebrae, sternum, bilateral multiple ribs, and pelvic bones. **(B)** The bone lesions in the left shoulder joint. **(C)** Bone lesions in the right hip joint. The red arrows in the figure indicate the lesions.

A 75-year-old woman complained of lower back pain after carrying a heavy case for 1 month. Computed tomography (CT) demonstrated multiple-site bone destruction, and bone cancer was considered. Immunofixation electrophoresis was carried out with the result of biclonal gammopathy (IgA-λ and IgG-λ) (**Figure 1**). She was then admitted to the Department of Hematology, Renji Hospital, Shanghai Jiao Tong University School of Medicine, on September 22, 2023. Her serum immunoglobulin quantification showed both increased IgA at 35.1 g/L (normal range: 1.0–4.2 g/L) and IgG at 23 g/L (normal range: 8.6–17.4 g/L). Two peaks in M protein were identified in serum protein electrophoresis, and the total quantity of M protein was 32.62 g/L, with M protein-1 at 22.63 g/L and M protein-2 at 9.99 g/L, respectively. She had no Bence-Joyce protein in her urine, and 24-h urine λ was 30.68 mg/L. Her serum free light chain (sFLC) revealed κ at 20.6 mg/L (normal range: 3.3–19.4 mg/L), λ at 154 mg/L (normal range: 5.7–26.3 mg/L), κ/λ ratio at 0.134, and κ-λ deviation of 133.4 mg/L. A complete blood count (CBC) showed anemia with hemoglobin at 82 g/L, but there were normal counts of white blood cells (WBCs) and platelets. Her peripheral blood smear revealed no plasma cells. She had normal creatinine and calcium. Serum albumin was 26.2 g/L (normal range: 40–55 mg/L), serum β2-microglobulin was 8 mg/L (normal range: 0.7–1.8 mg/L), and lactate dehydrogenase was 216 U/L (normal range: 120–250 mg/L). Bone marrow pathology showed active proliferation of bone marrow and obvious plasma cell proliferation with 31% mature plasma cells. Flow cytometry showed abnormal immunophenotype plasma cells accounting for 3.7082% of total nucleated cells and 98.84% of total plasma cells, which expressed CD38bri, CD138^+^, CD56^+^, CD19^−^, CD27^+^, CD117^−^, CD81^−^, and cLambda restricted expression. Among them, CD45^+^ abnormal plasma cells accounted for 1.04%, and CD45^−^ ones accounted for 2.67% of total nucleated cells. Bone marrow fluorescence *in situ* hybridization (FISH) showed only t(4;14)(p16.3;q32) 88.0% positive and 13q14 (RB1) 92.0% positive. PET-CT revealed multiple decreased bone density in the skull, multiple vertebrae, sternum, bilateral multiple ribs, and pelvic bones, some of which showed soft tissue density shadow filling. The diameter of the right hip lesion was 4.2 cm with increased FDG metabolism (SUVmax=8.7), consistent with the manifestation of MM involvement (**Figure 2**). Left calf muscle space mass with increased FDG metabolism (SUVmax=6.7) was detected by PET-CT, which was proven to be schwannoma by puncture biopsy. Then, this patient was diagnosed with MM (biclonal of IgG-λ and IgA-λ), Durie Salmon Staging System stage IIIA, ISS stage III, R-ISS stage III, R2-ISS stage III, and Mayo Stratification of Myeloma and Risk-Adapted Therapy (mSMART) high risk.

This patient had an Eastern Cooperative Oncology Group Performance Status (ECOG PS) score of 4 and a geriatric assessment (GA) score of 3, demonstrating her fragile status. The patient also had multiple comorbidities, including hypertension for over 10 years, lumbar disc protrusion, left lower limb deep venous thrombosis treated with edoxaban, and epilepsy secondary to surgery on cerebral hemorrhage due to cerebrovascular malformation and treated with levetiracetam. In addition, she was found with pneumonia with sputum and blood pathogen tests positive for cytomegalovirus, *Klebsiella pneumoniae*, *Acinetobacter baumannii*, *Stenotrophomonas maltophilia*, *Acinetobacter pitti*, and *Candida albicans*.

On September 26, 2023, the patient was treated with the bortezomib, cyclophosphamide, and dexamethasone (VCD) regimen as induction therapy (bortezomib 1.3 mg/m^2^ D1, 4, 8, 11 sc; cyclophosphamide 50 mg QD po D1-14, dexamethasone 20 mg D1-2, D4-5, D8-9, D11-12 iv) for MM. She also received symptomatic and supportive treatments such as denosumab to inhibit bone destruction, anti-infection (tigecycline, ceftazidine, ganciclovir, etc.), anticoagulation, and nutritional support. After one cycle of VCD, total M protein decreased from 32.62 to 2.4 g/L, indicating a very good partial response (VGPR). Pneumonia was ameliorated after anti-infection treatment.

Considering the patient’s age, performance status, comorbidities, and intolerance to additional chemotherapy, commercial equecabtagene autoleucel as first-line CAR-T therapy targeting BCMA was proposed and agreed upon by the patient and her family. For leukapheresis, the patient paused anti-MM therapy for 2 weeks after VCD treatment. Leukapheresis was carried out on October 23, 2023, on which day her WBC count was 5.01×10^9^/L, absolute neutrophil count was 3.81×10^9^/L, absolute lymphocyte count was 0.57×10^9^/L, hemoglobin (Hb) was 75 g/L, hematocrit was 23.7%, platelet (PLT) count was 351×10^9^/L, CD3^+^ T lymphocytes were 78.1%, and CD4/CD8 was 1.05. The cells were harvested (1.39×10^9^) and sent to the manufacturer for preparation of equecabtagene autoleucel. On November 15, 2023, the patient underwent a pre-lymphodepletion assessment, which demonstrated that she was still in VGPR with a total M protein of 1.29 g/L by protein electrophoresis, and her CBC returned to normal. On November 17, 2023, the fludarabine and cyclophosphamide (FC) regimen (fludarabine 25 mg/m^2^ D1-3, iv, and cyclophosphamide 250 mg/m^2^ D1-3, iv) was administered for lymphodepletion pre-treatment. In addition, paracetamol and diphenhydramine were administered 1 h before infusion to reduce the risk of infusion reactions. Two prescription doses of tocilizumab were handy for preventing cytokine release syndrome (CRS). Levetiracetam was increased to three tablets/day to avoid immune effector cell-associated neurotoxicity syndrome (ICANS).

On November 22, 2023 (day 0), the patient received transfusion of equecabtagene autoleucel. The final equecabtagene autoleucel product was positive for BCMA antigen-specific binding. Cell viability was 85%, the proportion and content of CD3^+^ CAR^+^ cells were 49.3% and 90%, and interferon (INF)-γ was 37 pg/mL. The proportion of CD4^+^ cells increased from 17.09% to 60.09% after post-collection sorting, and the proportion of CD8^+^ cells increased from 13.80% to 36.13%. The central memory T cells (Tcm) increased from 44.71% to 63.75%, effector memory T cells (Tem) decreased from 54.16% to 33.92%, and stem cell memory T cells (Tscm) increased from 0.69% to 2.15%. She was safely transfused with CAR-T cells at 1.0×10^6^/kg.

**Figure 3:**
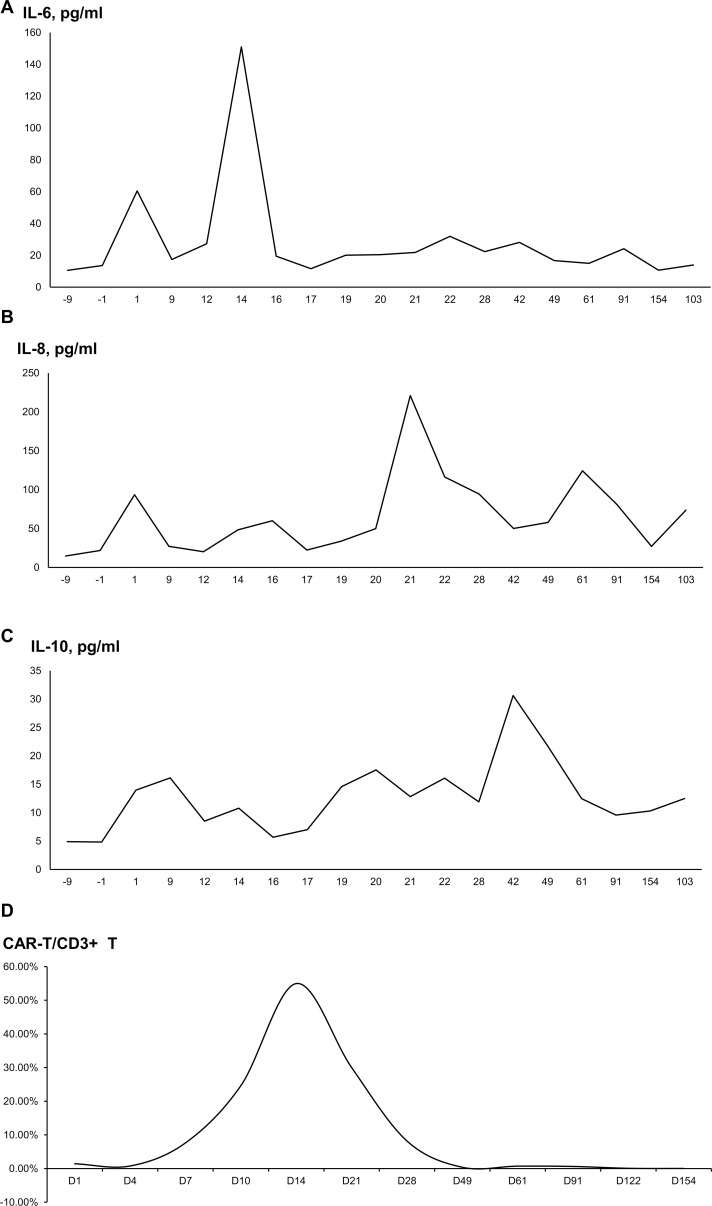
The proportion of cytokine levels and CAR-T cells/CD3^+^ T cells after CAR-T cell infusion. **(A)** Interleukin-6. **(B)** Interleukin-8. **(C)** Interleukin-10. **(D)** The proportion of CAR-T cells/CD3^+^ T cells.

After transfusion, the interleukin (IL)-6 and IL-8 levels were found gradually increased. IL-6 peaked at 150.98 pg/ml (normal range: 0–5.3 pg/mL) on day 14 and significantly decreased on day 16. IL-8 peaked at 220.94 pg/ml (normal range: 0–20.6 pg/mL) on day 21 and decreased on day 42 (**Figures 3A, B**). On the other hand, IL-10, INF-γ, and tumor necrosis factor-α showed no significant changes (**Figure 3C**). CRP gradually increased from 6.78 mg/L (normal range: 0–8 mg/L) to 13.31 mg/L, while procalcitonin (PCT) remained normal. A high-resolution chest CT examination showed an absorption of the pulmonary exudate. Intermittent administration of human immunoglobulin after CAR-T cell infusion was given to improve immunity. On day 14 after CAR-T cell infusion, the patient experienced a fever of 38.5°C, while blood pressure (BP) and oxygen saturation were normal. Rapid respiratory pathogen tests showed strong positive results for H1N1 influenza. Oseltamivir was administered for H1N1 influenza and nimesulide for fever reduction. Temperature returned to normal. The next morning, however, the patient developed restlessness, with SPO2 of 89%, BP of 80/40 mmHg, and heart rate of 180 beats/min. An electrocardiogram (ECG) showed rapid atrial fibrillation, which was suspected due to dehydration after antipyretics. The patient received nasal cannula oxygen inhalation therapy and was treated with norepinephrine for raising BP, and cedilanid and amiodarone hydrochloride for anti-atrial fibrillation. ECG monitoring demonstrated the heart rate gradually decreased to normal after treatment, and cardioversion was reached. WBC count and absolute neutrophil count declined to the lowest level of 1.85×10^9^/L and 1.32×10^9^/L on day 21, respectively. After treatment of G-CSF, her WBC count and absolute neutrophil count were recovered soon and stayed normal. Her Hb and PLT were in normal range with no change. Her increased cytokine levels and decreased WBC and neutrophils were considered related with H1N1 influenza infection. Neither CRS nor ICANS was observed in this patient.

On day 21 after infusion, an efficacy evaluation of MM was carried out. Protein electrophoresis showed no detectable M protein, and immunoelectrophoresis was negative for any monoclonal band. FLC returned to normal. BM smear showed 0.5% mature plasma cells, while MRD examination revealed no abnormal immunophenotypic plasma cells (minimal residual lesions <10^−6^) by EuroFlow cytometry, and her FISH for MM showed normal. She achieved sCR with MRD negativity, without other serious treatment-related toxicities or life-threatening conditions.

According to flow cytometry, the proportion of CAR-T cells/CD3^+^ T cells gradually increased from the infusion day. The proportion was 1.43% on day 1, peaking at 54.97% on day 14, and then gradually decreasing to 8.21% on day 28. The proportion was 0.07% on day 121 and 0.03% on day 153, which was still detectable (**Figure 3D**).

**Figure 4:**
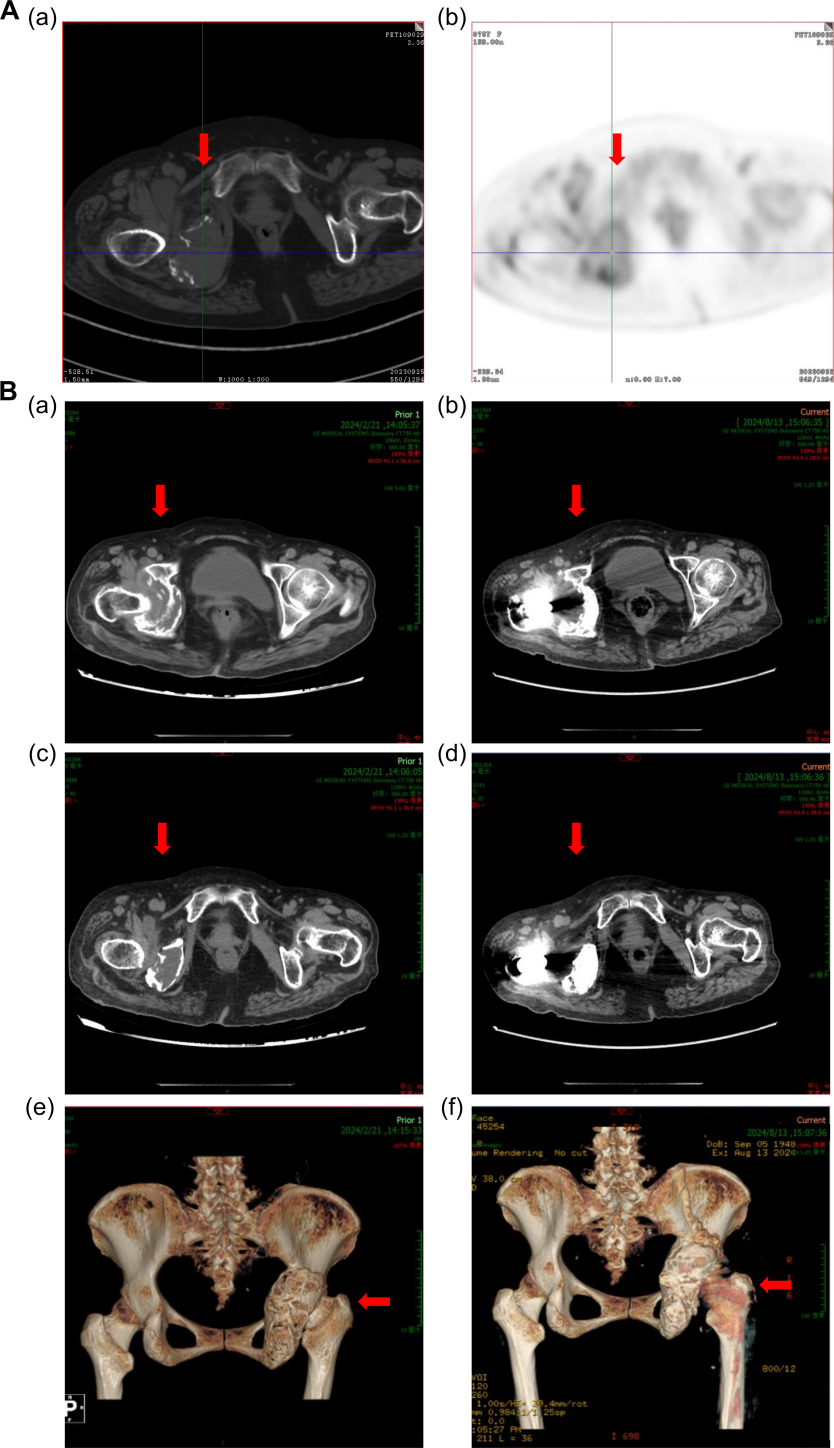
Computed tomography images of the right hip joint. **(A)** Computed tomography images show an extramedullary mass in the patient’s right hip joint in September 2023. **(B)** Reduction in the size of the extramedullary mass after 3 months of anti-BCMA CAR-T cell therapy, as shown in (a), (c), and (e) in February 2024. (c) Computed tomography images of the right hip joint after right artificial femoral head replacement, as shown in (b), (d), and (f) in August 2024. The red arrows in the figure indicate the lesions or surgical site.

In June of 2024, about 7 months following CAR-T cell infusion, the patient underwent right hip replacement plus pelvic lesion curettage due to pain in the right hip. The postoperative pathology indicated that there were no myeloma cells in the caput femoris bone tissue proven by immunohistochemistry staining. A hip joint CT scan was performed 2 months after the operation, which demonstrated that the size of the mass around the right caput femoris was decreased compared with that before CAR-T cell infusion, and callus formation was observed (**Figure 4**). And 3 months after the surgery, the patient was able to walk independently on flat ground. Hitherto, the patient has not received any further anti-MM treatment since the CAR-T cell infusion. As of the writing of this report, on December 11, 2024, the patient’s disease-free survival (DFS) has exceeded 12 months.

## Discussion

In this case, a 75-year-old fragile Chinese woman with biclonal and high-risk MM was reported to use anti-BCMA CAR-T cell treatment as first-line therapy. Biclonal MM is rare, with reported unfavorable outcome. She was stratified to the high-risk group in all established staging systems of MM, with positive t(4;14) and extramedullary mass in the right caput femoris. She also had multiple concomitant diseases. Considering her lower limb deep venous thrombosis, lenalidomide was not suitable for her due to its high risk of thrombosis. The PFS and OS benefit of daratumumab-containing regimens has been proven in frail MM patients (15, 16). However, daratumumab is not covered by medical insurance in China as first-line therapy for newly diagnosed MM patients. VCD regimen was then chosen for the patient as induction therapy. At the same time, she was administered with anti-infection therapy for her multiple microbial pulmonary infections. The patient achieved VGPR after one cycle of the VCD, and her pneumonia was improved. But she was evaluated too fragile to receive long-term conventional myeloma therapy.

Compared to the side effects of conventional myeloma therapy, such as bone marrow suppression and organ damage, CAR-T therapy has lower overall toxicity, and serious adverse reactions such as CRS can be effectively controlled through clinical management (17, 18). Furthermore, the modified CAR-T cells can continue to proliferate and exert anti-tumor effects *in vivo*, and MM patients may achieve long-term MRD-negative survival after CAR-T therapy, reducing the risk of frequent complications in conventional treatment (17, 18). Since no clinical trial of first-line CAR-T therapy was available for MM with ECOG PS 4 in China, commercial equecabtagene autoleucel, CAR-T therapy targeting BCMA, was proposed and supported by the patient and her family. Equecabtagene autoleucel (eque-cel), a fully human BCMA-targeted CAR-T cell therapy, was approved by NMPA in June 2023 for adult relapsed/refractory multiple myeloma patients after ≥3 prior lines of therapy. The pivotal FUMANBA-1 Ib/II clinical trial demonstrated significant efficacy and safety outcomes. Among 103 evaluable patients, the overall response rate reached 96.1% (99/103), with 80.8% (95% CI: 69.59–88.24) achieving sustained minimal residual disease (MRD) negativity for over 12 months. In the subgroup of 91 treatment-naïve patients to BCMA CAR-T therapy, the response rate further improved to 98.9% (90/91), accompanied by an 82.4% complete response/stringent complete response rate (75/91) and an 85.5% 12-month progression-free survival rate (95% CI: 75.75–91.51) (12–14). She was successfully treated with eque-cel, which achieved MRD-negative sCR without severe toxicity. A series of interesting observations were obtained from this case.

CAR-T cell therapy is approved for second-line treatment and above for MM patients, while limited evidence suggests their use as first-line therapy (17). However, relapses are common in the late-line CAR-T cell therapy (13, 18). T cell exhaustion is a cause of poor tumor control, and the maintenance of effector T cells after CAR-T treatment could also be involved in treatment response (17, 19–22). In the early stages of MM, T cells from patients are healthier and exhibit better fitness for CAR-T manufacturing, and the invasiveness of myeloma may be less extensive than those from relapsed/refractory patients (23, 24). Chemotherapy not only leads to T cell exhaustion but also leads to functional defects in surviving T cells. The applicability of CAR-T cells gradually decreases with the accumulation of chemotherapy lines (25). The KarMMa-3 (26) and CARTITUDE-4 (27) trials showed the potential of CAR-T cell therapy in earlier lines of treatment, and several trials are underway to demonstrate the effectiveness of CAR-T cell therapy as frontline therapy (NCT05695508, NCT05257083, and NCT05243797). Therefore, it is suggested that CAR-T cell treatment should be given in an earlier stage. Moreover, T cells should be collected from MM patients in the early stages of management, even for later use.

As for this patient, not only were the T cells collected in the early stage of the disease, CAR-T cell therapy was also administered as first line. The flow cytometry analysis showed that the ratio of CAR-T cells to CD3^+^ T cells gradually increased from the day of infusion, reaching a maximum of 54.97% on day 14 and then gradually decreasing, remaining at 0.03% on the 153rd day, indicating the long-term CAR-T cell survival. CAR-T cells also exhibited a high CD4/CD8 ratio, indicating the continued existence of CAR-T cell activity and long-term memory T cells. All of the above enlightened better therapeutic effects and promising survival in the patient. After BCMA CAR-T infusion, the patient’s MM efficacy evaluation on day 21 showed sCR with MRD negativity. Subsequently, she received right hip replacement plus pelvic lesion curettage 7 months after CAR-T transfusion; no myeloma cells were found in postoperative pathology. She remained sCR with MRD negativity up to the time of manuscript submission, which persisted for 12 months without any maintenance therapy. Accordingly, this report indicates that first-line anti-BCMA CAR-T cell therapy is effective for high-risk MM patients.

A real-world study showed that fragile patients with MM achieved good outcomes with CAR-T cell therapy and that toxicity was not more severe (28). The patient in this case was fragile with an ECOG score of 4 and a GA score of 3. She also had multiple comorbidities, such as pulmonary infection, secondary epilepsy, and lower limb venous thrombosis. The likelihood of adverse events such as CRS, ICANS, and severe hematological toxicity occurring during treatment had been predicted to be high in this patient. Before transfusion of equecabtagene autoleucel, paracetamol and diphenhydramine were given to reduce the risk of infusion reactions, levetiracetam was increased to prevent ICANS, tocilizumab was readied to prevent CRS, and the ICU was placed on standby if needed. Although the patient developed H1N1 influenza on day 14 and atrial fibrillation on day 15 after infusion, she recovered promptly and did not experience CRS, ICANS, or severe hematological toxicity throughout the treatment. This case proved that frailty does not constitute a contraindication to CAR-T cell therapy (29). Furthermore, it is noteworthy that the first-line CAR-T therapy given to the quite fragile patient appeared well tolerated and provided better life quality.

In conclusion, limited evidence supports the use of CAR-T cell therapy used as a first-line treatment in MM. However, this case demonstrated that first-line anti-BCMA CAR-T cell therapy was both effective and safe in a high-risk and fragile MM patient. The findings provide preliminary evidence supporting the advancement of CAR-T cell therapy to frontline MM treatment, warranting further evaluation in large-scale clinical trials.

## Data Availability

The original contributions presented in the study are included in the article/**Supplementary Material**. Further inquiries can be directed to the corresponding author.
