# Supplementary material for: First-line anti-BCMA CAR-T cell therapy in a fragile patient with biclonal gammopathy and giant plasma cell tumor multiple myeloma with multiple comorbidities: a case report
Source: Front Immunol. 2025 Apr 16;16:1564774. doi: 10.3389/fimmu.2025.1564774 (PMC12040831; doi:10.3389/fimmu.2025.1564774)
Supplement: Supplementary file 1 [file DataSheet1.pdf]

CARE case report guidelines

CARE Checklist of information to include when writing a case report

(cc) BY-NC-ND

Topic	Item	Checklist item description	Reported on Line
**Title**	1	The diagnosis or intervention of primary focus followed by the words "case report"	Line 0
**Key Words**	2	2 to 5 key words that identify diagnoses or interventions in this case report, including "case report"	Line 12-13
**Abstract (no references)**	3a	Introduction: What is unique about this case and what does it add to the scientific literature?	Line 15
	3b	Main symptoms and/or important clinical findings	Line 22
	3c	The main diagnoses, therapeutic interventions, and outcomes	Line 26
	3d	Conclusion—What is the main "take-away" lesson(s) from this case?	Line 61
**Introduction**	4	One or two paragraphs summarizing why this case is unique (**may include references**)	Line 64
**Patient Information**	5a	De-identified patient specific information.	Line 69
	5b	Primary concerns and symptoms of the patient.	LIne 77
	5c	Medical, family, and psycho-social history including relevant genetic information	Line 78
	5d	Relevant past interventions with outcomes	NA
**Clinical Findings**	6	Describe significant physical examination (PE) and important clinical findings.	Line 82
**Timeline**	7	Historical and current information from this episode of care organized as a timeline	LIne 82
**Diagnostic Assessment**	8a	Diagnostic testing (such as PE, laboratory testing, imaging, surveys).	Line 82
	8b	Diagnostic challenges (such as access to testing, financial, or cultural)	NA
	8c	Diagnosis (including other diagnoses considered)	Line 102-121
	8d	Prognosis (such as staging in oncology) where applicable	Line 109
**Therapeutic Intervention**	9a	Types of therapeutic intervention (such as pharmacologic, surgical, preventive, self-care)	Line122
	9b	Administration of therapeutic intervention (such as dosage, strength, duration)	Line 123/130/142/149/190
	9c	Changes in therapeutic intervention (with rationale)	NA
**Follow-up and Outcomes**	10a	Clinician and patient-assessed outcomes (if available)	Line 128/141/182/199
	10b	Important follow-up diagnostic and other test results	Line 177/198
	10c	Intervention adherence and tolerability (How was this assessed?)	Line 165
	10d	Adverse and unanticipated events	Line 165
**Discussion**	11a	A scientific discussion of the strengths AND limitations associated with this case report	Line 257
	11b	Discussion of the relevant medical literature **with references**.	Line 211/291
	11c	The scientific rationale for any conclusions (including assessment of possible causes)	Line 211
	11d	The primary "take-away" lessons of this case report (without references) in a one paragraph conclusion	LIne 257
**Patient Perspective**	12	The patient should share their perspective in one to two paragraphs on the treatment(s) they received	NA
**Informed Consent**	13	Did the patient give informed consent? Please provide if requested	Yes ☑ No ☐
